# Prognosis Analysis and Validation of Fatty Acid Metabolism-Related lncRNAs and Tumor Immune Microenvironment in Cervical Cancer

**DOI:** 10.1155/2022/4954457

**Published:** 2022-07-28

**Authors:** Xiaolin Lang, Changchang Huang, Hongyin Cui

**Affiliations:** Department of Gynecology, First People's Hospital of Linping District, Hangzhou, China

## Abstract

Cervical cancer (CC) is the third most common carcinoma and the fourth leading cause of cancer-associated mortality in women. The deregulation of fatty acid metabolism plays a crucial role in the progression of various tumors. This study is aimed at exploring the prognostic values of fatty acid metabolism- (FAM-) related long noncoding RNAs (lncRNAs) in CC. FAM-related differentially expressed genes (DEGs) and lncRNAs were screened in CC specimens based on TCGA datasets. Univariate analysis was carried out on differentially expressed lncRNAs to screen the survival-related lncRNAs. Multivariate assays were performed on the resulting lncRNAs to create a novel risk model. Survival assays were applied to examine the prognostic abilities of our model. Receiver operating characteristic (ROC) analysis was used to evaluate the accuracy of the new model. The association between risk model and immune responses was analyzed. In this study, we screened 9 differently expressed lncRNAs associated with the clinical outcome of CC patients. A nine-lncRNA signature comprising SCAT1, AC119427.1, AC009097.2, MIR100HG, AC010996.1, AL583856.2, MIAT, AP003774.2, and AC004540.2 was established to predict overall survival of CC. Survival assays revealed that patients' high risk score showed a shorter overall survival than those with low risk score. Multivariate assays demonstrated that the nine-gene signature was an independent prognostic factor in CC. In addition, we observed that APC_co_stimulation, CCR, and parainflammation were distinctly different between low-risk and high-risk groups. Our group observed a distinct difference in the expressions of CD44, TNFRSF8, CD276, LAG3, TNFRSF14, TMIGD2, VTCN1, TNFRSF25, CD80, NRP1, TNFRSF18, CD70, TNFSF9, and LGALS9 between the two groups of patients. Overall, our findings indicated that the 9 FAM-related lncRNA signature might be a promising prognostic factor for CC and can promote the management of FAM-related therapy in clinical practice.

## 1. Introduction

Cervical cancer (CC) is as one of the most common gynecological malignant tumors worldwide and has become a prominent public health issue [1]. The most recent global data to be released by the International Agency for Research on Cancer indicates that there were 569,847 newly diagnosed cases of cervical cancer in the world in 2018, and that the disease was responsible for the deaths of 311,365 people [2, 3]. The presence of a persistent infection with a high-risk human papillomavirus (HPV) is a primary determinant in the development of cervical cancer, but it is not a necessary condition [4]. Surgery, chemotherapy, and radiation therapy are the basic treatment options for patients who have been diagnosed with cervical cancer [5, 6]. In spite of the progress that has been made in medical technology, approximately one quarter of patients diagnosed with cervical cancer will either have a cancer recurrence or pass away over the next three years [7, 8]. Therefore, it is necessary to identify new prognostic markers and treatment options for CC to improve the survival of CC patients.

One of the 10 hallmarks of cancer is a metabolic disorder called dysregulation, and there is mounting evidence to suggest that metabolic reprogramming plays an important part in the beginning stages of cancer and its progression [9, 10]. In cancer cells, lipid metabolic reprogramming is one of the most notable metabolic alterations documented and has gained growing attention [11]. Malignant cells, in particular, take up a lot of glucose and glutamine to make proteins, lipids, and nucleic acids, all of which are necessary for cell proliferation [12, 13]. Fatty acids and other bioactive lipid compounds are altered by changing the lipid metabolism (FAs) [14]. For many years, researchers have known that FAs play a critical role in cancer cells' ability to synthesize membranes and provide energy during times of metabolic stress [15, 16]. In addition, the role of fatty acid metabolism in tumors has attracted renewed attentions as a major secondary messenger that contributes to tumor growth [17, 18]. However, nothing is known about how CC's fatty acid metabolic pathway is regulated. As a result, the discovery of genes associated to fatty acid metabolism may open up new therapy options for CC.

Moreover, seventy percent of gene transcripts are noncoding RNAs, which means that only 2% of genes can code for proteins [19]. A family of noncoding transcripts that are more than 200 nucleotides long has been referred to as long noncoding RNAs (lncRNA) [20]. Recent researches have shown that certain lncRNAs have an important role in the initiation, development, and outcome of several cancers [21, 22]. Normally, however, lncRNAs were deemed nonfunctional. lncRNAs that are linked to cancer progression can help scientists better understand the development of new treatments for the disease. A notion put forth by Salmena et al. in 2011 about competitive endogenous RNA (ceRNA), which has since been supported by a variety of studies [23]. It is hypothesized that some RNAs, known as ceRNAs, compete with one another for shared binding sites on target miRNAs, hence, changing their function. lncRNAs might influence FAM-related mRNA expressions in CC via sponging miRNAs, according to the theory. It is not known how FAM-related lncRNAs are regulated despite the fact that several CC ceRNA networks have been built in research.

In this study, using TCGA data, we first created a predictive multi-lncRNA signature for fatty acid metabolism-related lncRNAs. We then looked into the involvement of mRNA and immune responses related to fatty acid metabolism in CC prognosis.

## 2. Materials and Methods

### 2.1. Data Download and Preprocessing

TCGA database (https://cancergenome.nih.gov/) was used to download raw sequencing data and clinical information. Annotation and integration produced the standardized datasets. Integration processing of datasets was done using Perl scripting tools (http://www.perl.org/). A total of 309 samples were enrolled in this study, including 306 CC specimens and 3 matched normal tissues.

### 2.2. Differentially Expressed Gene Screening

Using the “limma” R package, we identified differentially expressed RNAs (referred to as DElncRNAs and FAM-mRNAs, respectively) based on the following criteria: ∣logfold change (FC) | >1 and false discovery rate (FDR) 0.05 [24].

### 2.3. Functional Enrichment Analysis of the Differentially Expressed Genes (DEGs) between CC Specimens and Nontumor Specimens

Filtering the DEGs between the CC samples and the normal samples was based on particular criteria (∣log2FC | >1 and FDR > 0.05). Analysis of Gene Ontology (GO) and the Kyoto Encyclopedia of Genes and Genomes (KEGG) pathways using the “clusterProfiler” program was carried out based on these DEGs.

### 2.4. Developments of a Novel Prognostic Signature Based on FAM-Related lncRNAs

We applied univariate and multivariate assays for the development of the FAM-related lncRNA signature, stratified based on risk score (Coefficient lncRNA1 × expressions of lncRNA1) + (Coefficient lncRNA2 × expressions of lncRNA2) + ⋯+(Coefficient lncRNAn × expressions of lncRNAn). Each CC patient's risk score was also assessed. It was determined by the median score that the RNAs may be classed as either low risk (less than the median number) or high risk (more than the median number).

### 2.5. Nomogram Construction

Combining genetic risk score values with clinical features, a nomogram was developed to predict 3- and 5-year CC OS. The nomogram's prediction power was evaluated using a calibration plot. For both the 3-year and 5-year OS ROC curves, the nomogram's sensitivity and specificity were analyzed using the AUC.

### 2.6. Immunity Analysis and Gene Expression

Comparing cell components or immune responses between low- and high-risk groups based on the FAM-related lncRNA signature, the TIMER, ssGSEA, MCPcounter, ESTIMATE, and CIBERSORT algorithms was also done [25–27]. Using a heat map, we were able to see the changes in immune response among the various methods. Besides, ssGSEA was employed to compare the immune cell subpopulations infiltrating tumors in the two groups and to gauge their immunological function. In addition, a possible immunological roadblock was found in previous research.

### 2.7. Statistical Analysis

R 3.5.3 was used to do all of the statistical analysis. *p* < 0.05 was chosen as the threshold for statistical significance. Using one-way ANOVA or Student's *t* test, the significance of variations in risk score and clinicopathological features was evaluated. Using Kaplan-Meier survival curves, researchers were able to compare survival rates between high-risk and low-risk groups of patients. Cox proportional hazard models were used to assess the hazard ratios of prognostic factors and to identify independent prognostic factors in the study.

## 3. Results

### 3.1. Identification of Differentially Expressed FAM-Associated Genes

**Figure 1:**
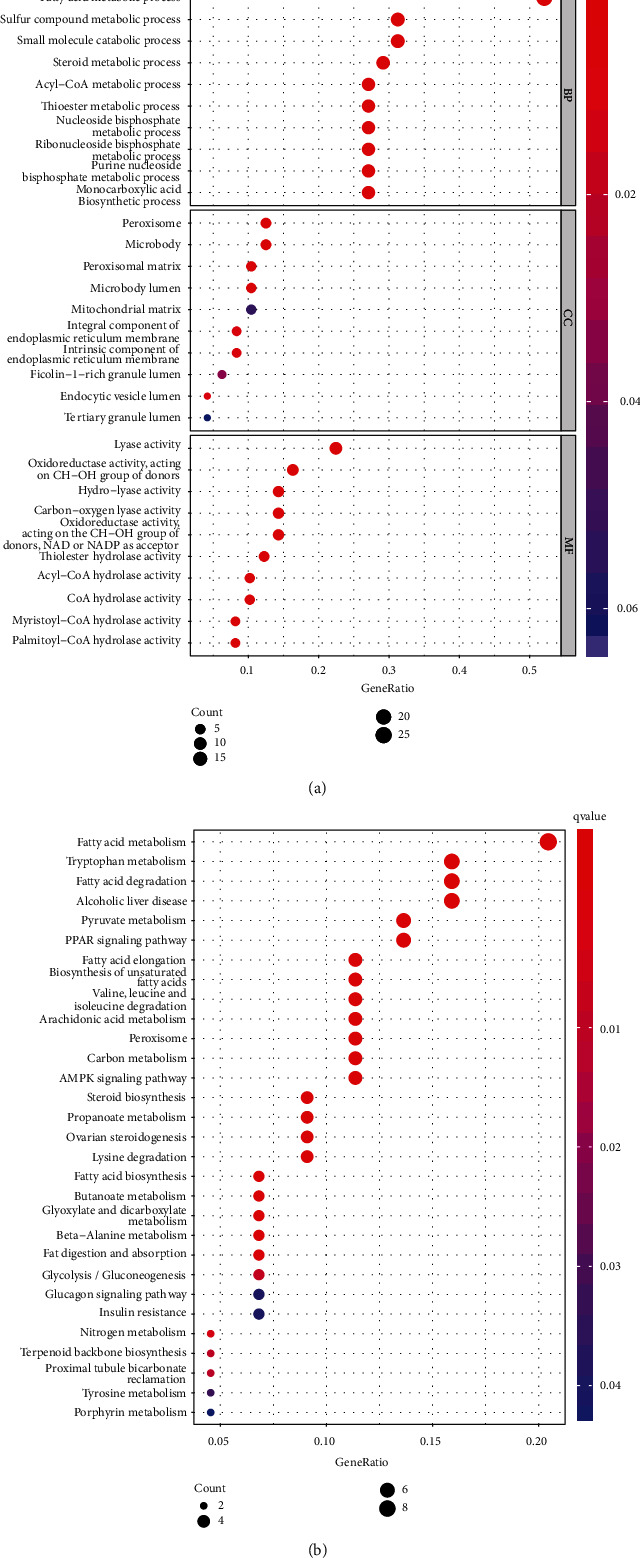
Functional enrichment analysis of the DEGs between CC specimens and nontumor specimens. (a) Distinctly enriched GO terms of DEGs in CC and (b) significant KEGG pathway terms of DEGs in CC.

We identified 49 FAM-associated genes (16 downregulated and 33 upregulated) that exhibited a dysregulated level in the TCGA datasets (Table S1). For the biology investigation, the 50 FAM-associated DEGs were applied for GO and KEGG enrichment. MF, BP, and CC were shown in Figure 1(a) with the top 10 phrases for each. In BP, the terms were predominantly related to fatty acid metabolic process, sulfur compound metabolic process, small molecule catabolic process, steroid metabolic process, and acyl-CoA metabolic process. In CC, the terms were primarily related to peroxisome, microbody, peroxisomal matrix, microbody lumen, and mitochondrial matrix. In MF, the terms were mainly related to lyase activity, hydro−lyase activity, acyl-CoA hydrolase activity, and CoA hydrolase activity. KEGG assays indicated the DEGs were mainly involved in fatty acid metabolism, tryptophan metabolism, fatty acid degradation, alcoholic liver disease, pyruvate metabolism, and PPAR signaling pathway (Figure 1(b)).

### 3.2. Construction of the lncRNA Prognostic Signature

**Figure 2:**
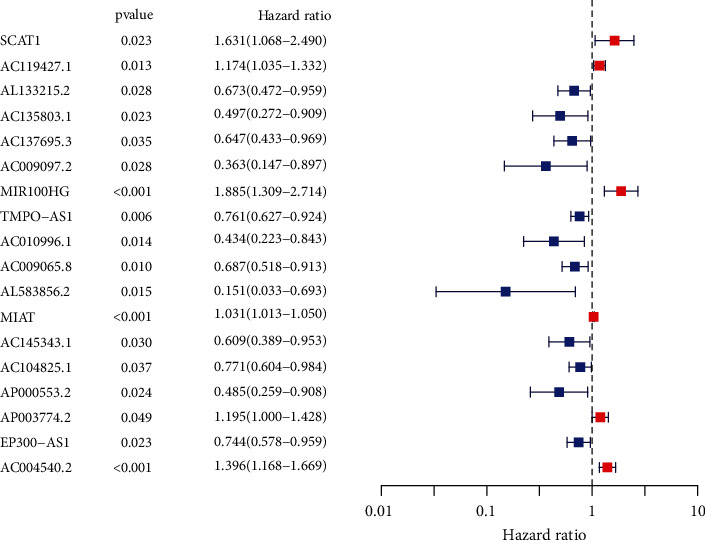
Univariate assays were applied to examine the survival-related FAM-related lncRNAs in CC.

**Figure 3:**
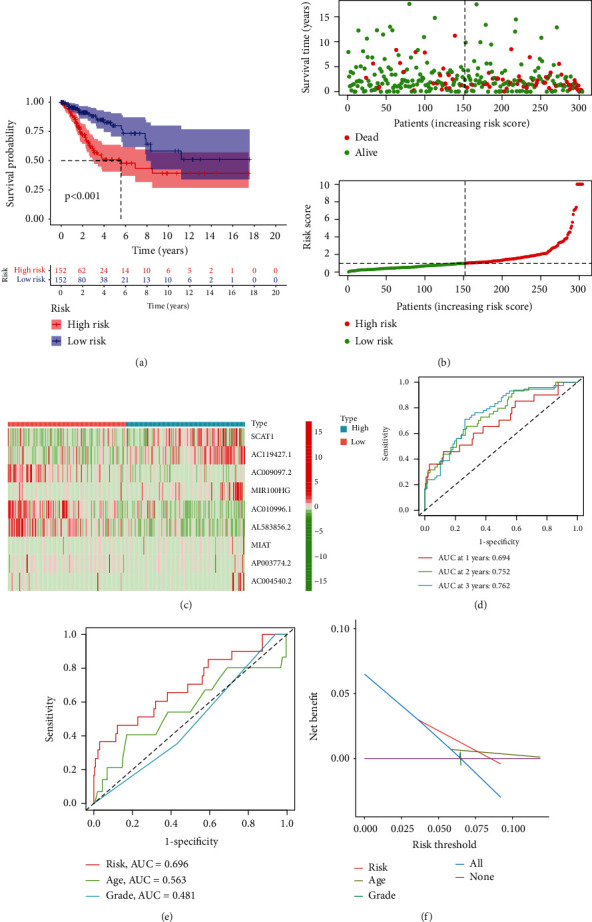
A novel prognostic model based on FAM-related lncRNAs for CC patients. (a) Kaplan-Meier assays revealed that patients in the high-risk group had a considerably shorter overall survival time. (b, c) Risk survival status plot. (d) ROC assays for 1, 3, and 5-year survivals of CC. (e) The AUC values of the risk factors. (f) The DCA of the risk factors.

**Figure 4:**
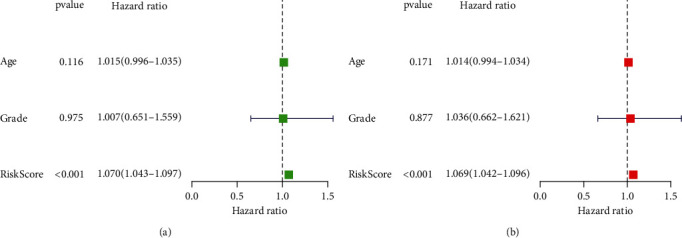
Univariate and multivariate assays of prognostic factors in CC patients.

**Figure 5:**
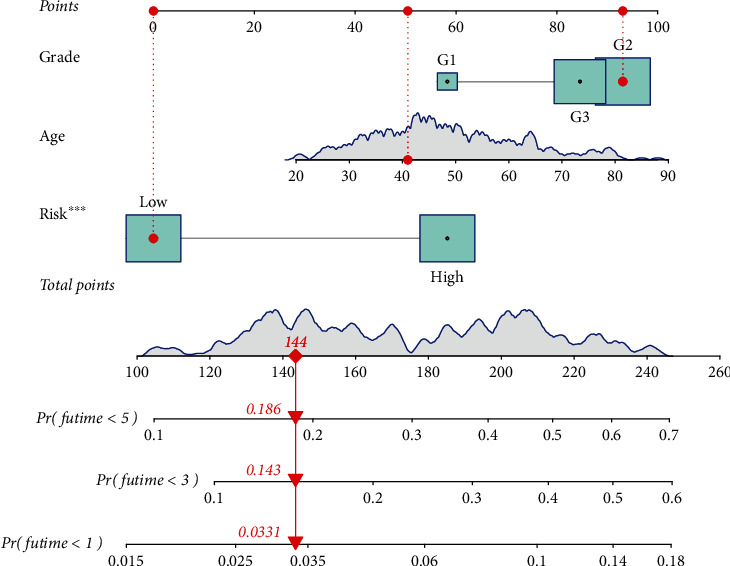
There is a nomogram for predictive FAM-related lncRNAs as well as for clinical factors. ∗∗∗*p* < 0.001.

Subsequently, we screened 124 FAM-related DElncRNAs. Then, we performed univariate COX analysis and identified 19 prognostic DElncRNAs in CC (Figure 2). Further, multivariate COX analysis identified nine lncRNAs (SCAT1, AC119427.1, AC009097.2, MIR100HG, AC010996.1, AL583856.2, MIAT, AP003774.2, and AC004540.2) as significant independent prognostic factors for CC patients (Table S2). The risk score of each case was calculated as follows: risk score = (0.605003 × expressions of SCAT1) + (0.242673 × expressions of AC119427.1) + (−0.97288 × expressions of AC009097.2) + (0.755143 × expressions of MIR100HG) + (−0.61929 × expressions of AC010996.1) + (−1.61362 × expressions of AL583856.2) + (0.027691 × expressions of MIAT) + (0.185731 × expressions of AP003774.2) + (0.207345 × expressions of AC004540.2). We used the median cutoff to divide the CC samples into high- and low-risk groups after rating each patient's risk via the signature. By performing a Kaplan-Meier survival analysis, we discovered that the OS for those in the high-risk group was much lower than that of those in the low-risk group (Figure 3(a)). Our results showed that most of new lncRNAs discovered in this research had a negative correlation with our risk model, which necessitates further investigation (Figures 3(b) and 3(c)). The unique lncRNA signature has an AUC of 0.694, 0.752, and 0.762 for predicting survival rates of 1, 3, and 5 years (Figure 3(d)). CC's prognosis was predicted by the characteristic lncRNAs with an AUC of 0.696, which was higher than the usual clinicopathological criteria (Figure 3(e)). The results of DCA also confirmed the prognostic value of novel model in CC patients (Figure 3(f)). Univariate and multivariate assays indicated that lncRNA signature was an independent prognosis factor of OS of CC patients (Figures 4(a) and 4(b)). Clinical care of CC patients can benefit from the hybrid nomogram that incorporates clinicopathological features and the unique FAM-related lncRNA prognostic signature (Figure 5).

### 3.3. Immunity and Gene Expression

**Figure 6:**
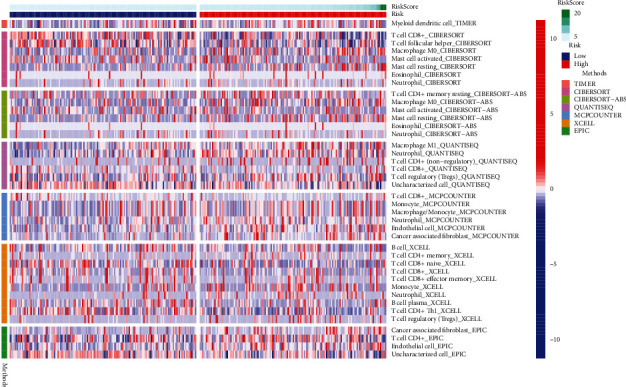
Heat map for immune responses by the use of TIMER, ssGSEA, MCPcounter, ESTIMATE, and CIBERSORT algorithms among low- and high-risk groups.

**Figure 7:**
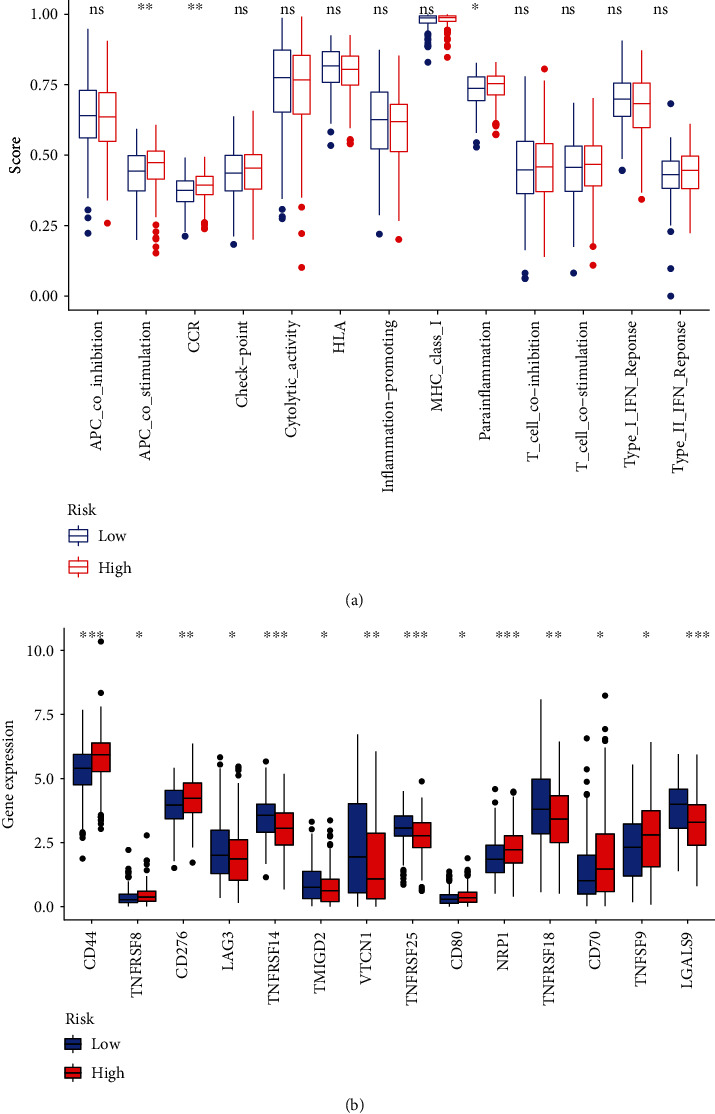
(a) ssGSEA for the relationship between related functions and immune cell subpopulations (b). Expressions of immune checkpoints among low and high CC risk groups. ∗∗∗*p* < 0.001, ∗∗*p* < 0.01, and ∗*p* < 0.05.

According to CIBERSORT, MCP counter, single-sample gene set enrichment (ssGSEA), and TiMER algorithms, the heat map of immune responses was depicted in Figure 6. According to data from TCGA datasets, ssGSEA study showed that APC costimulation, CCR, and parainflammation were distinctly different between low-risk and high-risk groups in terms of correlation analysis (Figure 7(a)). With regard to immunotherapy, we examined the difference in the expressions of immunological checkpoints between two groups. Our group observed a distinct difference in the expressions of CD44, TNFRSF8, CD276, LAG3, TNFRSF14, TMIGD2, VTCN1, TNFRSF25, CD80, NRP1, TNFRSF18, CD70, TNFSF9, and LGALS9 between the two groups of patients (Figure 7(b)).

## 4. Discussion

The prognosis of individuals with advanced or metastatic CC remains bleak despite the best treatment options available at the time of their diagnosis [28]. As the anticancer treatment of the future, immunotherapy may be an alternative for individuals with CC who are suffering from this condition [29]. FAM-related prognostic signatures are here for the first time identified, providing an effective prognostic model for CC patients and possible biomarkers of immunotherapy efficacy.

In this study, we screened 49 FAM-related DEGs. KEGG assays indicated the genes mainly participated in fatty acid metabolism, tryptophan metabolism, fatty acid degradation, alcoholic liver disease, pyruvate metabolism, and PPAR signaling pathway, suggesting these genes played an important role in fatty acid metabolism and the tumor progression. Then, we identified 124 fatty acid metabolism-related lncRNAs. After univariate and multivariate assays, we identified nine lncRNAs (SCAT1, AC119427.1, AC009097.2, MIR100HG, AC010996.1, AL583856.2, MIAT, AP003774.2, and AC004540.2) as distinctly independent elements for prognosis of CC patients. Some of the lncRNAs listed above have been linked to CC prognosis and progression in previous researches. For instance, MIR100HG expression was distinctly upregulated in early-stage CC and predicted a shorter overall survival [30]. Liu et al. reported that MIAT expression was distinctly decreased in CC and its overexpression proliferation of CC cells via modulating miRNA-150-5p [31]. In this study, we firstly created a prognostic signature using the nine fatty acid metabolism-related lncRNAs. We observed that patients with high risk score showed a shorter overall survivals. Importantly, univariate and multivariate assays suggested that lncRNA signature was an independent prognosis factor of overall survival of CC patients. Our findings suggested this novel signature may be applied as a novel prognostic biomarker for CC patients.

Nomograms are increasingly being utilized to evaluate tumor prognosis. As a result, nomograms can be used to tailor risk assessments to individual patients depending on their clinical or illness characteristics [32, 33]. A predictive nomogram integrating clinical characteristics and FAM-related lncRNA signals was created in this investigation. Patients with higher risk scores had shorter OS than patients with lower risk scores, according to the findings. The observed and anticipated rates at 1-, 3-, and 5-year intervals are perfectly consistent, according to the nomograms we developed. When comparing the observed and anticipated rates over time intervals of 1, 3, and 5, our nomograms demonstrate perfect agreement at all points in time. Based on 9 FAM-related lncRNAs, a risk model that can identify new biomarkers for follow-up investigations is quite reliable.

A variety of cancers have been found to be treatable by immunotherapy's ability to boost the immune system [34]. Preclinical and clinical studies show that immune-based therapies can improve the prognosis of patients with CC [35]. Moreover, immunotherapy in combination with other therapeutic approaches may also become a viable treatment option for CC. In addition, tumor-infiltrating lymphocytes, such as regulatory T cells and tumor-associated macrophages, have also been shown in recent investigations to be important in driving immune evasion during CC growth [36, 37]. In this study, high levels of myeloid dendritic cell, T cell CD8+, T cell follicular helper, macrophage M0, and mast cell activated were found in the high-risk group, suggesting immune tolerances in the patients with high risk score. Therefore, patient selection for more effective antitumor immunotherapies could be aided by the FAM-related lncRNA signature. In order to better recognize the role of the novel signature in predicting immunotherapeutic responses in CC patients, further validation is needed. Immune checkpoints were expressed at a higher level in patients at high risk in our research. In combination with checkpoint blockade, targeting tumor-specific ferroptosis pathways and possibly certain lncRNAs is a viable strategy for these patients. Our finding highlighted the potential of our model used as a novel indicator for immune response.

However, some limitations of this study should be noted. First, the prognostic model employed in this investigation was developed using data from a single source (TCGA). External, independent data sets and long-term follow-up should be used to confirm the prognostic value of our novel model in CC patients. Second, the Kaplan-Meier estimates of the TCGA cohort may be affected by the high censored rate. Third, explicit mechanisms should be elaborated in the further study.

## 5. Conclusion

We used publicly available databases to gather information and looked into the involvement of FAM-related lncRNAs in CC development. Based on nine FAM-related lncRNAs, we developed and validated a predictive risk signature that is reliable and sensitive. In addition, we developed a prognostic nomogram that could accurately predict the prognosis of CC patients. Patients with CC can benefit from the proposed signature in terms of immunotherapy and drug selection.

## Data Availability

The data used to support the findings of this study are included in the article. Data and materials from this study are available on request from the corresponding author.

## Supplementary Materials

Supplementary 1 Table S1: the fatty acid metabolism-related differentially expressed genes (DEGs) between CC specimens and normal specimens.

Supplementary 2 Table S2: multivariate analysis of overall survival in CC patients.
