# Supplementary material for: Prognosis Analysis and Validation of Fatty Acid Metabolism-Related lncRNAs and Tumor Immune Microenvironment in Cervical Cancer
Source: J Immunol Res. 2022 Jul 28;2022:4954457. doi: 10.1155/2022/4954457 (PMC9356243; doi:10.1155/2022/4954457)
Supplement: Supplementary 1 — Table S1: the fatty acid metabolism-related differentially expressed genes (DEGs) between CC specimens and normal specimens. [file 4954457.f1.docx]

Table S1 The Fatty Acid Metabolism-Related differentially expressed genes (DEGs) between CC specimens and normal specimens.

| gene | conMean | treatMean | logFC | pValue | fdr |
| --- | --- | --- | --- | --- | --- |
| CEL | 0.1766 | 14.59175 | 6.368524 | 0.005503 | 0.042866 |
| EHHADH | 1.133167 | 4.767423 | 2.07285 | 0.006322 | 0.042866 |
| ACACA | 3.1603 | 7.893601 | 1.320622 | 0.007986 | 0.04561 |
| CA4 | 2.078333 | 0.12847 | -4.01593 | 0.00352 | 0.042866 |
| ACOT7 | 7.036467 | 20.04936 | 1.510633 | 0.009481 | 0.048657 |
| HACD2 | 8.0167 | 24.0068 | 1.582363 | 0.006839 | 0.042866 |
| CYP1B1 | 13.89667 | 2.885702 | -2.26774 | 0.005394 | 0.042866 |
| CPOX | 5.3228 | 11.84834 | 1.154428 | 0.007834 | 0.04561 |
| HSD17B11 | 39.91237 | 9.101156 | -2.13271 | 0.003978 | 0.042866 |
| CYP2U1 | 3.7531 | 0.966355 | -1.95746 | 0.005841 | 0.042866 |
| HSPH1 | 7.203767 | 20.61377 | 1.516785 | 0.006448 | 0.042866 |
| ALDH1B1 | 36.86557 | 10.8514 | -1.76439 | 0.009303 | 0.048657 |
| DHCR24 | 9.272033 | 96.84361 | 3.384699 | 0.003231 | 0.042866 |
| HCCS | 6.209433 | 16.1425 | 1.37833 | 0.003231 | 0.042866 |
| CYP2J2 | 0.181867 | 3.222138 | 4.147065 | 0.006199 | 0.042866 |
| THEM5 | 0.4402 | 8.406879 | 4.255339 | 0.004977 | 0.042866 |
| CPT1B | 0.017067 | 0.177156 | 3.375766 | 0.004829 | 0.042866 |
| TDO2 | 0.030567 | 0.517436 | 4.081348 | 0.009661 | 0.048657 |
| ACOT11 | 0.246267 | 1.117574 | 2.182077 | 0.007392 | 0.045234 |
| FASN | 5.908233 | 30.9506 | 2.389169 | 0.005503 | 0.042866 |
| CA2 | 0.4502 | 38.25233 | 6.408838 | 0.003164 | 0.042866 |
| AOC3 | 46.76643 | 1.424439 | -5.03701 | 0.003164 | 0.042866 |
| ACAT1 | 10.7866 | 3.740417 | -1.52797 | 0.004878 | 0.042866 |
| GABARAPL1 | 31.18987 | 11.66282 | -1.41916 | 0.004977 | 0.042866 |
| NSDHL | 10.6745 | 28.13953 | 1.398429 | 0.00344 | 0.042866 |
| ACAT2 | 3.6846 | 12.94716 | 1.813056 | 0.009481 | 0.048657 |
| ACBD7 | 0.112033 | 2.339592 | 4.384257 | 0.005958 | 0.042866 |
| H2AZ1 | 51.9816 | 134.7071 | 1.373753 | 0.004407 | 0.042866 |
| ALOX12B | 0.0141 | 2.289837 | 7.343406 | 0.008378 | 0.046808 |
| ALDOA | 0.446533 | 3.637399 | 3.026068 | 0.00478 | 0.042866 |
| ACOT4 | 0.6079 | 3.016749 | 2.311089 | 0.009845 | 0.048657 |
| SERINC1 | 123.2994 | 41.78988 | -1.56094 | 0.003033 | 0.042866 |
| TECR | 13.80233 | 33.89617 | 1.29621 | 0.007684 | 0.04561 |
| NUDT19 | 3.576767 | 9.774717 | 1.450399 | 0.005079 | 0.042866 |
| PCCB | 2.8459 | 7.102708 | 1.319484 | 0.006706 | 0.042866 |
| SCD | 5.575067 | 106.041 | 4.249489 | 0.003033 | 0.042866 |
| PTGIS | 69.04097 | 0.884258 | -6.28684 | 0.003098 | 0.042866 |
| INMT | 9.256667 | 0.498379 | -4.21518 | 0.003231 | 0.042866 |
| ACACB | 4.963967 | 1.131175 | -2.13367 | 0.003299 | 0.042866 |
| ADH1B | 13.9245 | 0.156475 | -6.47555 | 0.002973 | 0.042866 |
| ACOX2 | 5.036467 | 0.427505 | -3.5584 | 0.003369 | 0.042866 |
| LTC4S | 4.090033 | 1.592944 | -1.36042 | 0.006839 | 0.042866 |
| LGALS1 | 1057.759 | 239.811 | -2.14104 | 0.004318 | 0.042866 |
| SLC27A2 | 0.105133 | 3.539789 | 5.073371 | 0.004498 | 0.042866 |
| HSP90AA1 | 130.3589 | 287.4534 | 1.140839 | 0.005841 | 0.042866 |
| DBI | 16.157 | 69.41313 | 2.103049 | 0.002908 | 0.042866 |
| GPD2 | 3.351067 | 13.92785 | 2.05528 | 0.003098 | 0.042866 |
| IDH1 | 8.041667 | 25.75904 | 1.679512 | 0.005614 | 0.042866 |
| HSD17B7 | 1.198833 | 2.617403 | 1.126505 | 0.005726 | 0.042866 |
