# Supplementary material for: Prognosis Analysis and Validation of Fatty Acid Metabolism-Related lncRNAs and Tumor Immune Microenvironment in Cervical Cancer
Source: J Immunol Res. 2022 Jul 28;2022:4954457. doi: 10.1155/2022/4954457 (PMC9356243; doi:10.1155/2022/4954457)
Supplement: Supplementary 2 — Table S2: multivariate analysis of overall survival in CC patients. [file 4954457.f2.docx]

Table S2 Multivariate analysis of overall survival in CC patients.

| id | coef | HR | HR.95L | HR.95H | pvalue |
| --- | --- | --- | --- | --- | --- |
| SCAT1 | 0.605003 | 1.831258 | 1.189868 | 2.818386 | 0.005956 |
| AC119427.1 | 0.242673 | 1.274652 | 1.108615 | 1.465556 | 0.000654 |
| AC009097.2 | -0.97288 | 0.377994 | 0.146455 | 0.97559 | 0.04432 |
| MIR100HG | 0.755143 | 2.127916 | 1.36452 | 3.318402 | 0.000866 |
| AC010996.1 | -0.61929 | 0.538329 | 0.2693 | 1.076116 | 0.079708 |
| AL583856.2 | -1.61362 | 0.199165 | 0.040933 | 0.969069 | 0.045621 |
| MIAT | 0.027691 | 1.028078 | 1.000664 | 1.056243 | 0.044632 |
| AP003774.2 | 0.185731 | 1.204099 | 1.046808 | 1.385024 | 0.00931 |
| AC004540.2 | 0.207345 | 1.230408 | 0.95925 | 1.578214 | 0.10259 |
